# Leaf epidermal micromorphology defining the clades in *Cinnamomum* (Lauraceae)

**DOI:** 10.3897/phytokeys.182.67289

**Published:** 2021-10-04

**Authors:** Zeng Gang, Bing Liu, Jens G. Rohwer, David Kay Ferguson, Yong Yang

**Affiliations:** 1 College of Biology and Environment, Nanjing Forestry University, 159 Longpan Road, Nanjing 210037, Jiangsu, China Institute of Botany, the Chinese Academy of Sciences Beijing China; 2 State Key Laboratory of Systematic and Evolutionary Botany, Institute of Botany, the Chinese Academy of Sciences, 20 Nanxincun, Xiangshan, Beijing 10093, China Xishuangbanna Tropical Botanical Garden, Chinese Academy of Sciences Mengla China; 3 Xishuangbanna Tropical Botanical Garden, Chinese Academy of Sciences, Mengla 666303, China Universität Hamburg Hamburg Germany; 4 Universität Hamburg, Institute of Plant Science and Microbiology, Ohnhorststr. 18, 22609 Hamburg, Germany University of Vienna Vienna Austria; 5 University of Vienna, Department of Paleontology, 1090 Vienna, Austria Nanjing Forestry University Vienna Austria

**Keywords:** Anatomy, *Cinnamomum*, Lauraceae, scanning electron microscope (SEM), systematics

## Abstract

In this study, we sampled 48 species of Asian *Cinnamomum* covering the species groups that were identified in recent phylogenetic studies and conducted leaf micromorphological observations using both light microscopy (LM) and scanning electron microscopy (SEM). Synapomorphies were determined by means of mapping micromorphological characters on a phylogenetic tree. The results indicate that *Cinnamomum* exhibits two different types of leaf upper epidermis: Type I has smooth/non-reticulate periclinal walls whereas Type II has reticulate periclinal walls and is unusual in the family Lauraceae. We found that the two types of micromorphological characters are clade-specific, sect. Camphora s.s. possesses Type I leaf upper epidermis, and sect. Cinnamomum s.l. has Type II leaf upper epidermis. Our study also reveals that *C.saxatile*, a member of sect. Camphora s.l. in the traditional classification, actually has Type II leaf upper epidermis, thus reinforcing the result of a recent molecular phylogeny that has this species in a clade consisting mainly of species of sect. Cinnamomum.

## Introduction

In the family Lauraceae, there are some named generic complexes according to molecular systematic studies, e.g. the *Beilschmiedia* group, the *Persea* group, the *Litsea* group, the *Alseodaphne* group, and the *Cinnamomum* group (e.g. Chanderbali et al. 2001; Rohwer et al. 2009, 2014; Huang et al. 2016; Trofimov et al. 2016, 2019; Mo et al. 2017; Rohde et al. 2017; Trofimov and Rohwer 2020; Xiao et al. 2020; Liu et al. 2021). A number of macromorphological characters have been used in the past to define the genera in each complex, but it now seems that these macromorphological characters were either plesiomorphic (e.g. in *Ocotea* Aubl. s.l.) or originated through parallelism, i.e. evolved several times. For instance, in the *Litsea* group, *Lindera* Thunb. differs from *Litsea* Lam. in the number of anther locules (2-locular in *Lindera* vs. 4-locular in *Litsea*). Phylogenetic studies based on DNA sequences suggest that *Lindera* is polyphyletic, and comprises many different clades (Li et al. 2004; Fijridiyanto and Murakami 2009). How these clades can be recognized using morphological characters has become an important question in the taxonomy of the group.

The *Cinnamomum* group is amphi-Pacific and distributed in tropical America and tropical to subtropical Asia with relatively few species found in Africa and Australia (Rohwer 1993; van der Werff 2001). The group belongs to the Laureae-Cinnamomeae clade of the core Lauraceae (Chanderbali et al. 2001; Rohwer and Rudolph 2005; Song et al. 2017, 2020), and consists of several closely related genera, i.e. *Cinnamomum* Schaeff., *Aiouea* Aubl. and the *Ocotea* complex (Chanderbali et al. 2001; Huang et al. 2016; Rohde et al. 2017). The group is thought to have originated ca. 55 mya and was once widely distributed in the palaeotropical Arcto-Tertiary flora of Laurasia during the Eocene, then migrated southwards and, with cooling temperatures, split, resulting in the modern amphi-Pacific disjunct distribution (Huang et al. 2016).

**Figure 1.**
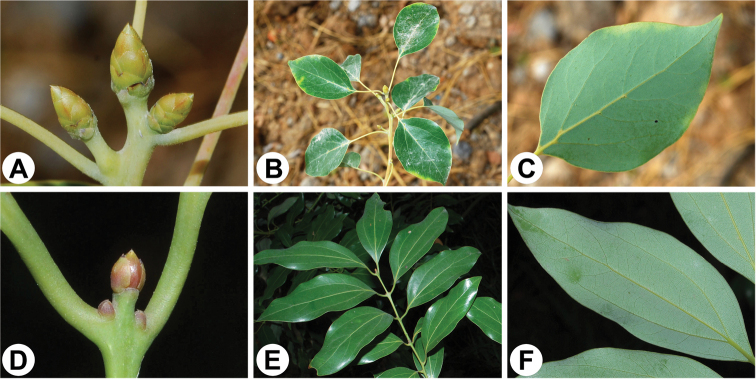
Morphology of the two sections of the Asian *Cinnamomum***A–C***Cinnamomumcamphora* of sect. Camphora**A** perulate terminal buds **B** branch portion displaying the alternate leaf arrangement **C** a leaf showing the pinnate venation and the domatia in axils of lateral veins **D, E***Cinnamomumjaponicum* of sect. Cinnamomum**D** terminal buds lacking helically arranged scales **E** branch portion exhibiting the subopposite leaf arrangement **F** a leaf displaying the tripliveined venation and the absence of domatia in axils of lateral veins.

*Cinnamomum* is generally considered to consist of ca. 300 species, with the highest diversity in tropical Asia (Rohwer 1993; van der Werff 2001) and only a few species in Australia (Hyland 1989) and some Pacific Islands. Species of the genus are characterized by inflorescence Type II of van der Werff and Richter (1996), i.e. paniculate inflorescences with strictly opposite ultimate cymes, flowers with nine fertile stamens plus three staminodia with a conspicuous cordate to sagittate glandular head, and a more or less developed cupule with or without persistent (remnants of) tepals (van der Rohwer 1993; van der Werff 2001). Traditionally, the Asian species have been classified into two sections: *Camphora* Meisn. (1864: 24) and *Cinnamomum*. Species in sect. Camphora have alternate leaves, usually with domatia in the axils of lateral veins, pinnate to subtriplinerved venation, and often perulate buds. Species in sect. Cinnamomum have (sub)opposite and tripliveined leaves lacking domatia in the axils of lateral veins, and no perulate buds (Fig. 1, e.g. Li et al. 1982). Recent phylogenetic studies have consistently suggested that Asian *Cinnamomum* is not monophyletic and contains two robust clades (Huang et al. 2016; Trofimov and Rohwer 2020). Cinnamomumsect.Camphora s.s. (Clade 1) excluding three species of the traditional sect. Camphora, and sect. Cinnamomum s.l. (Clade 2) containing three species previously attributed to sect. Camphora s.l. (*C.saxatile* H.W. Li (1975: 44), *C.longipetiolatum* (Gamble) N. Chao ex H.W. Li (1975: 47), and an unidentified species labelled *C.* sp. C684; Huang et al. 2016). The species of the Neotropical clade (Clade 3) recognized by Huang et al. (2016) have recently been transferred to *Aiouea* (Rohde et al. 2017), as the result of a study of nrITS sequences and two chloroplast spacers (*psb*A-*trn*H and *trn*G-*trn*S).

Various studies have suggested different topologies, e.g. sect. Cinnamomum s.l. as sister to the Neotropical clade in Huang et al. (2016), but sect. Camphora s.s. appears to be sister to the Neotropical clade in the ITS result of Rohde et al. (2017). According to Rohde et al. (2017), sect. Camphora s.s. and the Neotropical clade share alternate, penninerved to moderately triplinerved leaves. Huang et al. (2016) suggested that alternate and penninerved leaves, perulate buds and domatia are potential synapomorphies for sect. Camphora s.s., but these characters do not seem to be very reliable (Huang et al. 2016). Though Trofimov and Rohwer (2020) confirmed that Asian *Cinnamomum* is diphyletic, they gave different relationships of the two sections of the Asian *Cinnamomum*: sect. Camphora appears to be sister to *Sassafras* J. Presl, and together they constitute a clade which appears to be sister to the Neotropical Ocotea complex, whereas sect. Cinnamomum is sister to *Kuloa* Trofimov & Rohwer, an African genus recently described. Liu et al. (2021) found pervasive conflicts between plastid data and nrITS, and suggested that Asian *Cinnamomum* was paraphyletic with respect to *Sassafras*. They included only a single Neotropical sample from the Cinnamomeae, *Nectandraangustifolia* (Schrad.) Nees et Mart. (1833: 48). In any case, Asian *Cinnamomum* contains two robust clades. However, it remains unclear how the clades of *Cinnamomum* should be defined morphologically.

Leaf epidermal micromorphology has been considered to be of taxonomic importance within the Lauraceae (Christophel et al. 1996; Nishida and Christophel 1999; Nishida and van der Werff 2007, 2011, 2014; Yang et al. 2012; Zeng et al. 2014; Nishida et al. 2016; Trofimov and Rohwer 2018), but its systematic significance has rarely been discussed within a phylogenetic context. In this study, we report micromorphological observations in Asian *Cinnamomum*, and discuss their systematic significance.

## Material and methods

Mature leaf materials were taken from herbarium specimens. Our sampling covered the two clades that were identified in recent molecular phylogenetic studies (Huang et al. 2016; Rohde et al. 2017), and contained 48 species of Asian *Cinnamomum*. Three *Sassafras* species were also sampled for comparison because the result based on *psb*A-*trn*H and *trn*G-*trn*S sequences in Rohde et al. (2017) suggested that *Sassafras* was closely related to at least some species of *Cinnamomum*.

Leaf samples were obtained from herbarium specimens deposited in the Herbarium of the State Key Laboratory of Systematic and Evolutionary Botany, Institute of Botany (PE), the Chinese Academy of Sciences (Table 1). Earlier studies have suggested that leaf epidermal characters are stable within species (e.g. Nishida and Christophel 1999; Nishida and van der Werff 2007, 2011; Yang et al. 2012). Therefore, in most cases, one specimen was sampled to represent the species in this study. For the purpose of comparison and to eliminate variation that might be caused by sampling from different leaf areas, we took samples close to the basal portion on the left hand side of the midvein of a leaf. The leaf materials were then cut into small rectangular pieces (ca. 3 mm×10 mm).

**Table 1.** Voucher information of leaf samples for this study.

Latin Name	Collection	Locality
*Cinnamomumappelianum*Schewe (1925: 20)	S.H. Chun 10175	China, Guangxi
*C.aromaticum*Nees (1831b: 74)	Anshun Exped. 049	China, Guizhou
*C.austrosinense* H.T. Chang (1959: 20)	S.Y. Chang 3133	China, prov. unknown
*C.bejolghota* (Buch.-Ham.) Sweet (1826: 344)	S.Z. Cheng & B.S. Li 03985	China, Xizang
*C.bodinieri* H. Lév. (Léveillé 1912: 369)	P.C. Tsoong 34	China, Guizhou
*C.burmannii* (Nees & T. Nees) Blume (1826: 569)	G.Z. Li 15650	China, Guangxi
*C.camphora* (L.) J. Presl (1825: 47)	S.Y. Chang 4819	China, Zhengjiang
*C.camphora*	L.D. Duan 2601	China, Hunan
*C.camphora*	C.F. Liang 33262	China, Guangxi
*C.chartophyllum* H.W. Li (1975: 49)	B. Liu 1366	China, Yunnan
*C.chekiangense*Nakai (1939: 23)	H. Zou 01435	China, Anhui
*C.daphnoides* Siebold & Zucc. (Siebold and Zuccarini 1846: 402)	T. Yahara 6641	Japan, Kyushu
*C.doederleinii* Engl. (Engler 1884: 57)	M. Furuse 43512	Japan, Kyushu
*C.glanduliferum* (Wall.) Meisn. (Meisner 1864: 25)	Y.M. Shui 2217	China, Yunnan
*C.ilicioides* A. Chev. (Chevalier 1918: 855)	F.C. How 72957	China, Hainan
*C.iners* (Reinw. ex Nees et T. Nees) Blume (1826: 570)	Y. Tsiang 12772	China, Yunnan
*C.insularimontanum*Hayata (1913: 158)	T.Y.A. Yang et al. 08378	China, Taiwan
*C.japonicum*Siebold (1830: 23)	Zhejiang Bot. Exped. 27696	China, Zhejiang
*C.jensenianum* Hand.-Mazz. (Handel-Mazzetti 1921: 63)	T.T. Yü 3125	China, Sichuan
*C.liangii*C.K. Allen (1939: 58)	S.K. Lau 26252	China, Hainan
*C.litseifolium*Thwaites (1861: 253)	M. Poilane 14784	Cambodia
*C.longipaniculatum* (Gamble) N. Chao ex H.W. Li (1975: 48)	Z.W. Yao 3567	China, Sichuan
*C.macrostemon*Hayata (1913: 160)	Y.H. Lai 27	China, Taiwan
*C.mairei* H. Lév. (Léveillé 1914: 174)	Z.W. Yao 4980	China, Yunnan
*C.micranthum* (Hayata) Hayata (1913: 160)	M. Poilane 10707	Cambodia
*C.migao* H.W. Li (1978: 90)	Beijing Team 891144	China, Guangxi
*C.osmophloeum* Kaneh. (Kanehira 1917: 428)	C.M. Wang 05395	China, Taiwan
*C.ovalifolium* Gardner ex Meisn. (Meisner 1864: 11)	T. Koyama 13513	Sri Lanka
*C.parthenoxylon* (Jack) Meisn. (Meisner 1864: 26)	Sichuan Bot. Exped. 2355	China, Sichuan
*C.parthenoxylon*	IBCAS Team. 814	China, Jiangxi
*C.pauciflorum*Nees (1831b: 75)	Mt. Ziyun Exped. 411	China, Hunan
*C.pedunculatum* (Thunb.) J. Presl (1825: 37)	Jiangxi Exped. 947	China, Jiangxi
*C.pingbienense* H.W. Li (1978: 91)	B. Liu 1363	China, Yunnan
*C.pittosporoides* Hand.-Mazz. (Handel-Mazzetti 1925: 19)	Yunnan Exped. of CAS 311	China, Yunnan
*C.pseudopedunculatum*Hayata (1913: 161)	M. Furuse 52925	Japan, Kyushu
*C.randaiense*Hayata (1911: 238)	T.Y. Liu et al. 201	China, Taiwan
*C.reticulatum*Hayata (1911: 239)	G.F. Zhong et al. 1374	China, Taiwan
*C.rigidissimum* H.T. Chang (1959: 19)	C.F. Wei 122561	China, Hainan
*C.saxatile* H.W. Li (1975: 44)	B. Liu 1327	China, Yunnan
*C.scortechinii*Gamble (1910: 219)	M. Poilane 11143	Unknown locality
*C.septentrionale* Hand.-Mazz. (Handel-Mazzetti 1936: 213)	H. Yu 177	China, Sichuan
*C.subavenium* Miq. (Miqeul 1858: 902)	W.C. Cheng 3649	China, Zhejiang
*C.subavenium*	M.J. Wang 3768	China, Anhui
*C.tamala* (Buch.-Ham.) T. Nees & Eberm. (Nees 1831a: 2)	Qinghai-Xizang Veg. Exped. 4584	China, Xizang
*C.tenuifolium* (Makino) Sugim. (Sugimoto 1928: 57)	M. Furuse 8173	Japan
*C.tetragonum* A. Chev. (Chevalier 1918: 855)	M. Poilane 378	Unknown locality
*C.tonkinense* (Lecomte) A. Chev. (Chevalier 1918: 856)	Liu Bing 1326	China, Yunnan
*C.tsangii* Merr. (Merrill 1934: 26)	Jiangxi Exped. 124	China, Jiangxi
*C.validinerve*Hance (1882: 80)	X.G. Li 200631	China, Guangdong
*C.verum* J. Presl (1825: 37)	N. Wallich 2573B	Unknown locality
*C.wilsonii*Gamble (1914: 66)	Z.C. Luo 191	China, Hunan
*C.zollingeri* Lukman. (Lukmanoff 1889: 4)	S. Saito 1388	Japan, Nagato
*Sassafrasalbidum* (Nutt.) Nees (1836: 490)	S.C. Chen et al. 715	China, Taiwan
*S.randaiense* (Hayata) Rehder (1920: 244)	W.M. Wang 93	USA, Georgia
*S.tzumu* (Hemsl.) Hemsl. (Hemsley 1907: 55)	B. Liu 1372	China, Yunnan

*All specimens examined in this study are deposited in PE.

For light microscopic observation, the samples were dipped in 40% NaClO at 60 °C until the samples began to bleach. The samples were then washed in distilled water. The epidermis of both surfaces of the leaves was peeled off under a light microscope (Zeiss Stemi 2000), then stained in 1% safranin-50% ethanol for 30 minutes, dehydrated with gradations of ethanol, and treated with gradations from ethanol to xylene, and finally the epidermis pieces were mounted in Canada balsam (Yang et al. 2012; Zeng et al. 2014). The preparations were dried at 40 °C in an incubator. Photographs were taken using a Zeiss Axio Imager A1 light microscope with a 10× eyepiece and 40× objective.

For SEM observations, leaf samples were cut into small pieces of ca. 3 × 3 mm. Leaf samples were soaked in 100% ethanol for 15 minutes, followed by ultrasonic cleaning for 10 minutes at 100 hz, after which the ethanol was replaced by isoamyl acetate, and critical-point dried using carbon dioxide for five hours (equipment: HCP-2; Yang et al. 2012; Zeng et al. 2014). The treated leaf pieces were then fixed on stubs with the inner surface of leaf epidermis exposed, coated with palladium under 15 mA for 110 s, observed and photographed under a HITACHI s-4800 scanning electron microscope (10.0KV; State Key Laboratory of Systematic and Evolutionary Botany, Institute of Botany, the Chinese Academy of Sciences). To clarify the ornamentation of the upper leaf epidermis, the peeled upper leaf epidermis was also observed.

Observed features of leaf epidermis included the following: (1) epidermal cell shape, (2) anticlinal walls of normal epidermal cells, i.e. non-stomatal cells, (3) periclinal walls of normal epidermal cells, and (4) the stomatal complex (including subsidiary cells). Line-drawings were made with Adobe Photoshop CS2 ver. 9.0 using the Pen Tool.

Because our aim was to understand the evolution of micromorphological characters in the context of phylogeny, we selected published sequences according to our sampling for the micromorphological studies to reconstruct a phylogeny. *Phoebezhennan*, *P.hungmoensis*, *Alseodaphnopsisrugosa*, and *A.hainanensis* were selected as the outgroup for phylogenetic analysis of *Cinnamomum*. Micromorphology of these outgroup species was observed but has not been published yet (Zeng 2018). All sequences were downloaded from NCBI (Table 2). We used three nuclear genes to reconstruct the phylogeny of the Asian *Cinnamomum*, i.e. nrITS, *LEAFY* intron II, and *RPB*2. Sequences were aligned with MAFFT v7.304b (Katoh and Standley 2013). MrModeltest 2.3 (Nylander 2008) and Paup* v.4.0b10 (Swofford 2003) were used to select the best-fit evolutionary model using Akaike Information Criterion (AIC). Bayesian inference (BI) analysis was performed using MrBayes 3.2.6 on XSEDE (Huelsenbeck and Ronquist 2001). The Markov Chain Monte Carlo (MCMC) algorithm was run for 3,000,000 generations, sampling one out of every 1000 generations. The first 25% trees were discarded as burn-in. The remaining trees were used to calculate the posterior probabilities (PP) and construct the consensus tree. Maximum Likelihood (ML) analyses were performed using RAxML-HPC2 on XSEDE with the GTRCAT model to search the best-scoring ML tree and generate a tree block at the same time. 1000 bootstrap replicates were performed in each analysis to obtain the confidence support. The ML tree block was read in FigTree v1.4.0 and saved as a nexus file which was then opened in Mesquite v3.04. Micromorphological characters were collected based on leaf anatomy in this study, and were manually input into the “Character Matrix” in Mesquite. All characters were treated as unordered and equally weighted. To reconstruct character evolution, a maximum likelihood approach using Markov k-state 1 parameter model (Mk1; Lewis 2001) was used. We selected the “Trace-Character-Over-Trees” command to calculate ancestral states at each node including probabilities in the context of likelihood reconstructions. To carry out these analyses, characters were plotted onto trees that were sampled in ML analyses. The results were finally summarized as percentage of changes of character states on a given branch among the stored trees utilizing the option of “Average-frequencies-across-trees”. Trees with reconstructed ancestral character states were then exported as pdf files which were then manually adjusted in Adobe Illustrator CS6.

**Table 2.** Sequences obtained from the GenBank for phylogeny of Asian *Cinnamomum*.

Taxon	ITS	RPB2	LEAFY
*Alseodaphnopsishainanensis* (Merr.) H.W. Li & J. Li (2016: e0186545 (9))	FJ755440	KU140409	HQ697006
*A.rugosa* (Merr. & Chun) H.W. Li & J. Li (2016: e0186545 (9))	HQ697183	KU140410	HQ697012
*Cinnamomumappelianum*	KU139817	KU140330	KU140244
*C.austrosinense*	KU139818	KU140331	KU140245
*C.bejolghota*	KU139822	KU140335	KU140249
*C.bodinieri*	KU139824	KU140336	KU140251
*C.burmannii*	KU139825	KU140337	KU140252
*C.camphora*	KU139826	KU140338	KU140253
*C.chartophyllum*	KU139832	KU140344	KU140259
*C.chavarrianum* (Hammel) Kosterm. (Kostermans 1988: 442)	AF272261	KU140345	–
*C.chekiangense*	MF110041	KU140346	KU140260
*C.daphnoides*	FM957803	KU140352	KU140266
*C.doederleinii*	KU139842	–	KU174408
*C.glanduliferum*	KU139843	KU140354	KU140269
*C.iners*	KU139849	KU140360	KU140275
*C.insularimontanum*	KY271510	KU140361	KU174418
*C.japonicum*	KU139851	KU140361	KU140277
*C.jensenianum*	KU139853	KU140363	KU140279
*C.liangii*	KU139856	KU140366	KU174422
*C.longipaniculatum*	KX546754	KT248715	KU140283
*C.macrostemon*	GU598521	–	–
*C.mairei*	KU139859	KU140368	KU174423
*C.micranthum*	KY271519	KU140369	GQ260581
*C.osmophloeum*	KY271528	KU140375	–
*C.parthenoxylon*	KU139871	KU140377	KU140295
*C.pauciflorum*	KU139872	KU140378	KU140296
*C.pingbienense*	KU139873	KU140379	KU140297
*C.pittosporoides*	KU139874	KU140380	KU140298
*C.reticulatum*	KU139879	–	KU174432
*C.rigidissimum*	KU139881	KU140386	KU140305
*C.saxatile*	KU139882	KU140387	KU140306
*C.septentrionale*	KU139883	KU140388	KU140307
*C.subavenium*	KU139888	KU140393	KU140312
*C.tamala*	KX822090	KU140396	KU174439
*C.tenuifolium*	KU139892	KU140397	KU140316
*C.tenuifolium*	–	–	KU140316
*C.tonkinense*	KU139895	KU140400	KU140319
*C.tsangii*	KU139900	KU140405	KU140324
*C.verum*	MF110061	KU140407	KU140326
*C.wilsonii*	KU139904	KU140408	KU140328
*Phoebehungmoensis* S.K. Lee (1963: 190)	HQ697206	KU140413	HQ697138
*P.zhennan* S.K. Lee & F.N. Wei (1979: 61)	HQ697212	KT248761	HQ697161

*Note 1: these species were not included in phylogenetic studies, but belong to Clade I according to their non-reticulate periclinal walls. *Note 2: these species lack phylogenetic information, should belong to the clade II according to their reticulate periclinal walls. #*C.saxatile* is traditionally ascribed to sect. Camphora. Invisible refers to those species possessing thick trichomes or appendages covering the stomata.

## Results

**Figure 2.**
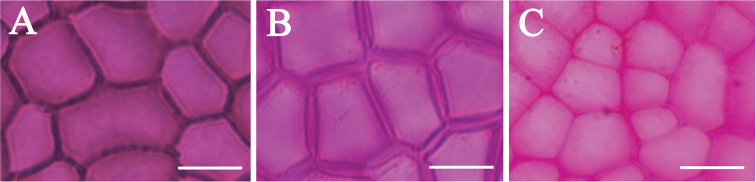
Leaf epidermal micromorphology of the Asian *Cinnamomum*, displaying the Type I upper leaf epidermis with polygonal cell shape, periclinal walls lacking reticulate ornamentations, and variable thickness of anticlinal walls **A***C.parthenoxylon***B***C.inunctum***C***C.bodinieri*. Bars: 20 μm.

**Figure 3:**
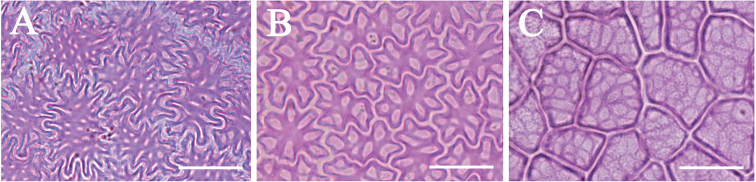
. Leaf epidermal micromorphology of *Cinnamomum*, displaying the Type II upper leaf epidermis with irregular or polygonal cell shape, reticulate periclinal walls, and straight, sinuous to extremely sinuous anticlinal walls **A***C.iners***B***C.appelianum***C***C.pittosporoides*. Scale bars: 20 μm.

**Figure 4.**
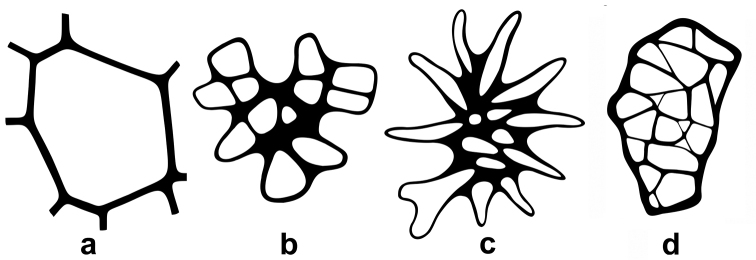
Line-drawing displaying variation of the leaf upper epidermis **a** type I, displaying the non-reticulate periclinal wall, the polygonal cells, and the round to polygonal cell shape **b** type II, displaying the sinuous anticlinal wall, the irregular cell shape, and the reticulate periclinal wall **c** type II, displaying an extremely sinuous anticlinal wall, the irregular cell shape, and the reticulate periclinal wall **d** type II, displaying the straight or nearly so anticlinal wall, the polygonal cell shape, and the reticulate periclinal wall.

Leaf epidermal micromorphology is presented in Figs 2, 3. Illustrations in Fig. 4 display main characters and their variation. The main results are presented in Table 3.

**Table 3.** Micromorphology of the leaf epidermis of Asian *Cinnamomum* under light microscopy (LM) and scanning electron microscope (SEM).

Clade	Latin name	Cell shape	Periclinal wall	Anticlinal wall	Lower stomatal ledge (LM)	Stomatal surface (SEM)	Section
**Clade 1**	*C.bodinieri*	polygonal	non-reticulate	straight/rounded	wide lip-shaped	eyelid-shaped	Sect. Camphora
	*C.camphora*	polygonal	non-reticulate	straight/rounded	wide lip-shaped or bat-shaped	globose	
	*C.chartophyllum*	polygonal	non-reticulate	straight/rounded	narrow lip-shaped	circular	
	*C.glanduliferum*	polygonal	non-reticulate	straight/rounded	wide lip-shaped or bat-shaped	globose	
	*C.longepaniculatum*	polygonal	non-reticulate	straight/rounded	bat-shaped	lip-shaped	
	*C.micranthum*	polygonal	non-reticulate	straight/rounded	wide lip-shaped	circular	
	*C.parthenoxylon*	polygonal	non-reticulate	straight/rounded	wide lip-shaped	globose	
	*C.septentrionale*	polygonal	non-reticulate	straight/rounded	wide lip-shaped	globose	
**Note 1**	*C.ilicioides*	polygonal	non-reticulate	straight/rounded	wide lip-shaped	eyelid-shaped	
	*C.migao*	polygonal	non-reticulate	straight/rounded	narrow lip-shaped	lip-shaped	
**Clade 2**	*C.appelianum*	irregular	reticulate	Sinuous	wide lip-shaped or bat-shaped	eyelid-shaped	Sect. Cinnamomum
	*C.austrosinense*	irregular	reticulate	Sinuous	wide lip-shaped or bat-shaped	eyelid-shaped	
	*C.bejolghota*	irregular	reticulate	Sinuous	-	globose	
	*C.burmannii*	irregular	reticulate	Sinuous	butterfly-shaped	eyelid-shaped	
	*C.cassia*	irregular	reticulate	Sinuous	butterfly-shaped	eyelid-shaped	
	*C.chekiangense*	irregular	reticulate	Sinuous	butterfly-shaped	eyelid-shaped	
	*C.iners*	irregular	reticulate	Sinuous	wide lip-shaped	eyelid-shaped	
	*C.insularimontanum*	irregular	reticulate	Sinuous	wide lip-shaped or butterfly-shaped	eyelid-shaped	
	*C.japonicum*	irregular	reticulate	Sinuous	wide lip-shaped or butterfly-shaped	eyelid-shaped	
	*C.jensenianum*	irregular	reticulate	Sinuous	butterfly-shaped	eyelid-shaped	
	*C.mairei*	irregular	reticulate	Sinuous	wide lip-shaped	invisible	
	*C.osmophloem*	irregular	reticulate	Sinuous	wide lip-shaped or butterfly-shaped	eyelid-shaped	
	*C.pauciflorum*	irregular	reticulate	Sinuous	butterfly-shaped	eyelid-shaped	
	*C.pedunculatum*	irregular	reticulate	Sinuous	wide lip-shaped or butterfly-shaped	eyelid-shaped	
	*C.pingbienense*	irregular	reticulate	Sinuous	wide lip-shaped	eyelid-shaped	
	*C.randaiense*	irregular	reticulate	Sinuous	wide lip-shaped	eyelid-shaped	
	*C.rigidissimum*	irregular	reticulate	Sinuous	wide lip-shaped	eyelid-shaped	
	#***C.saxatile***	irregular	reticulate	Sinuous	wide lip-shaped	eyelid-shaped	
	*C.subavenium*	irregular	reticulate	Sinuous	wide lip-shaped	eyelid-shaped	
	*C.tamala*	irregular	reticulate	Sinuous	wide lip-shaped or butterfly-shaped	lip-shaped	
	*C.tenuifolium*	irregular	reticulate	Sinuous	wide lip-shaped or butterfly-shaped	eyelid-shaped	
	*C.tonkinense*	irregular	reticulate	Sinuous	wide lip-shaped or bat-shaped	eyelid-shaped	
	*C.tsangii*	irregular	reticulate	Sinuous	wide lip-shaped	invisible	
	*C.verum*	irregular	reticulate	Sinuous	wide lip-shaped or narrow lip-shaped	eyelid-shaped	
	*C.wilsonii*	irregular	reticulate	Sinuous	wide lip-shaped or butterfly-shaped	eyelid-shaped	
	*C.zollingeri*	irregular	reticulate	Sinuous	wide lip-shaped	eyelid-shaped	
	*C.daphnoides*	polygonal	reticulate	straight/rounded	butterfly-shaped	invisible	
	*C.doederleinii*	polygonal	reticulate	straight/rounded	wide lip-shaped	eyelid-shaped	
	*C.pittosporoides*	polygonal	reticulate	straight/rounded	wide lip-shaped	globose	
	*C.reticulatum*	polygonal	reticulate	straight/rounded	bat-shaped	eyelid-shaped	
	*C.scortechinii*	polygonal	reticulate	straight/rounded	butterfly-shaped	globose	
*Note 2	*C.liangii*	irregular	reticulate	Sinuous	wide lip-shaped	eyelid-shaped	Sect. Cinnamomum
	*C.litseifolium*	irregular	reticulate	Sinuous	narrow lip-shaped	eyelid-shaped	
	*C.macrostemon*	irregular	reticulate	Sinuous	butterfly-shaped	eyelid-shaped	
	*C.pseudopedunculatum*	irregular	reticulate	Sinuous	wide lip-shaped	eyelid-shaped	
	*C.tetragonum*	irregular	reticulate	Sinuous	wide lip-shaped	eyelid-shaped	
	*C.validinerve*	polygonal/irregular	reticulate	undulate/rounded	wide lip-shaped	eyelid-shaped	
	*C.ovalifolium*	polygonal/irregular	reticulate	undulate/rounded	wide lip-shaped	eyelid-shaped	

*Note 1: these species were not included in phylogenetic studies, but belong to Clade I according to their non-reticulate periclinal walls. *Note 2: these species lack phylogenetic information, should belong to the clade II according to their reticulate periclinal walls. #*C.saxatile* is traditionally ascribed to sect. Camphora. Invisible refers to those species possessing thick trichomes or appendages covering the stomata.

### Micromorphology

**Figure 5.**
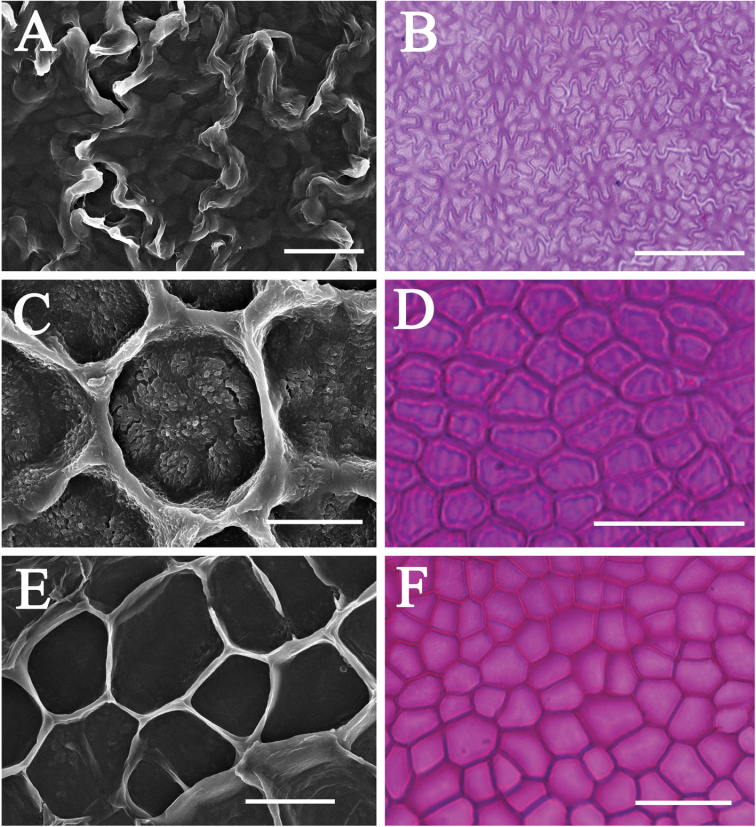
A comparison between the upper leaf epidermis under scanning electron microscope and light microscope, SEM images of internal surface of the upper leaf epidermis displaying the possible origin of the reticulations of the periclinal walls **A, B***C.aromaticum***C, D***C.daphnoides***E, F***C.ilicioides*. Scale bars: 10 μm; (**A, C**); 20 μm; (**E**); 50 μm (**B, D, F**).

The upper leaf epidermal micromorphology of Asian *Cinnamomum* species falls clearly into two types according to the reticulation of the periclinal walls of leaf upper epidermis, cell shape and straightness of anticlinal walls. Type I is characterized by polygonal epidermal cells, the anticlinal walls being straight or nearly so, the periclinal walls smooth and not reticulate (Figs 2, 4a). This type of epidermal cell is rather homogeneous. Variation occurs in the thickness of the anticlinal walls, e.g. not thickened, somewhat beaded (Fig. 2A), prominently thickened (Fig. 2B), or more or less thickened (Fig. 2C). Thickening of the anticlinal walls was based on visual perception and no measurements were made. Type II possesses epidermal cells with irregular outlines, with the anticlinal walls undulate to sinuous, and the periclinal walls reticulate (Figs 3, 4b, d). The cell shape and anticlinal walls are variable in straightness. In most species, the epidermal cells have an irregular shape, the anticlinal walls being either sinuous (Fig. 4b) or extremely sinuous, almost stellate in appearance (Fig. 4c). In a few species, the epidermal cells are polygonal and the anticlinal walls are straight or curved, e.g. *C.daphnoides*, *C.doederleinii*, *C.pittosporoides*, *C.reticulatum*, *C.scortechinii*, *C.validinerve*, and *C.ovalifolium*. The reticulation of the periclinal walls of the Type II results from the uneven thickening of the periclinal walls (Fig. 5A–D). In Type I, the periclinal walls are evenly thickened (Fig. 5E, F). The leaf epidermal characters such as the anticlinal wall straightness, cell shape and periclinal wall ornamentation are stable within species and not influenced by external stimuli.

**Figure 6.**
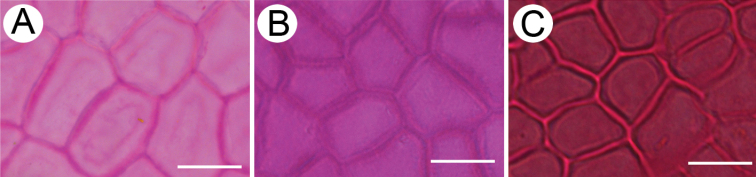
Leaf epidermal micromorphology of *Sassafras* displaying the non-reticulate periclinal wall in the genus **A***S.albidum***B***S.randaiense***C***S.tzumu*. Scale bars: 20 μm (**A–C**).

The leaf micromorphology of *Sassafras* is presented in Fig. 6. The upper leaf surface is very similar to Type I of *Cinnamomum*, the epidermal cells being rectangular or polygonal, the anticlinal walls straight or nearly so, and the periclinal walls not reticulate (Fig. 6A–C). The stomata are elliptic in outline, with reniform subsidiary cells, or lip-shaped with narrower subsidiary cells, the subsidiary cells usually raised. The periclinal walls of epidermal cells are slightly wrinkled and immersed in *S.albidum*, but raised in *S.tzumu* and *S.randaiense*.

**Figure 7.**
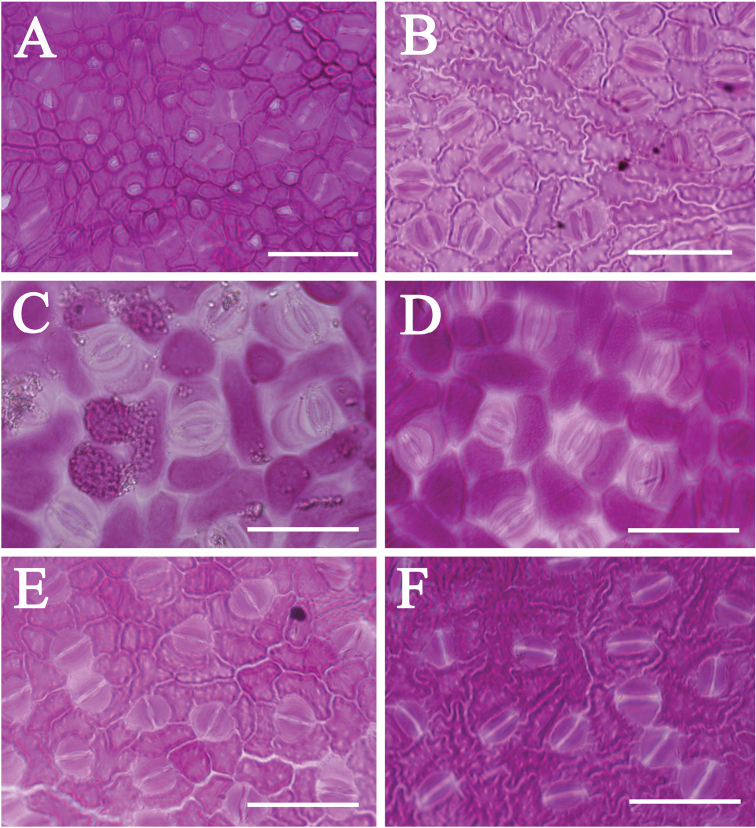
Lower leaf epidermis of *Cinnamomum* under light microscope (LM) **A***C.daphnoides* displaying butterfly-shaped stomata **B***C.burmannii* displaying butterfly-shaped stomata **C**C.migao displaying narrow lip-shaped stomata **D***C.longepaniculatum* displaying bat-shaped stomata **E***C.randaiense* displaying narrow lip-shaped stomata **F***C.verum* displaying wide lip-shaped/wide lip-shaped stomata. Scale bars: 50 μm.

**Figure 8.**
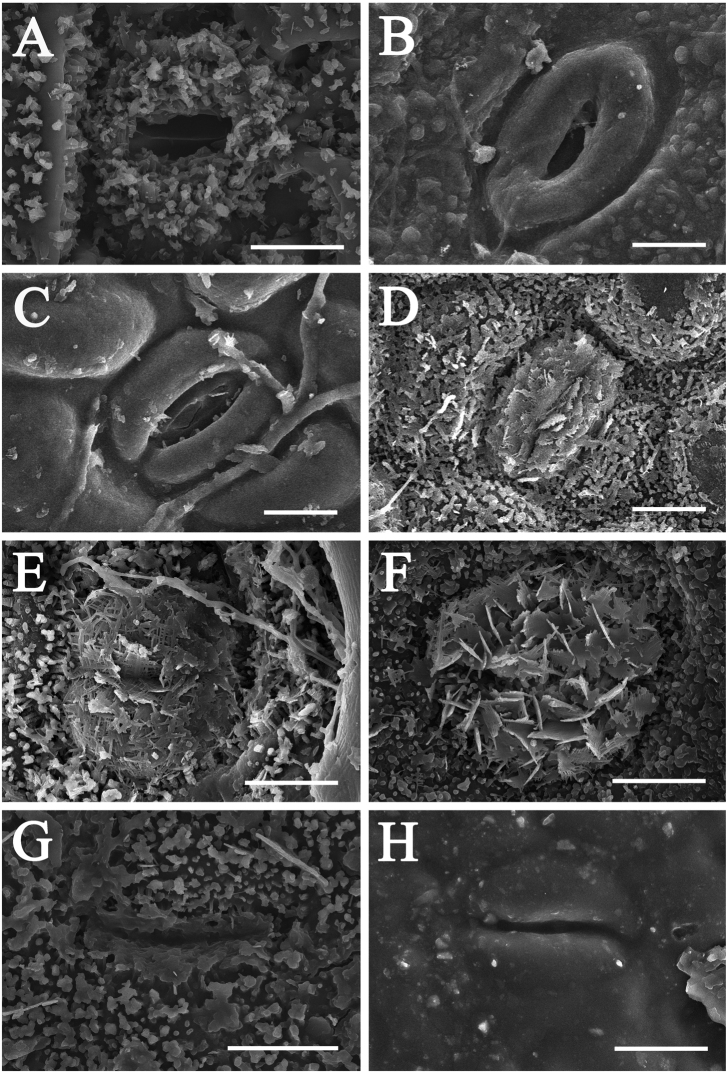
Lower leaf epidermis of *Cinnamomum* under scanning electron microscope displaying stomatal features **A, B** circular stomata **A***C.chartophyllum***B***C.micranthum***C, D** lip-shaped stomatal **C***C.migao***D***C.longepaniculatum***E, F** globose stomata **E***C.septentrionale***F***C.camphora***G, H** eyelid-shaped stomata **G***C.tonkinense***H***C.jensenianum*. Scale bars: 10 μm.

Lower leaf epidermis comprises epidermal cells and stomata. Epidermal cells are polygonal (e.g. *C.camphora*, *C.daphnoides*, and *C.glanduliferum*), round (e.g. *C.bodinieri*, *C.glanduliferum*, *C.migao*, and *C.porrectum*) or irregular/amoeboid in shape (e.g. *C.burmannii*, *C.randaiense*, *C.saxatile* and *C.subavenium*). Anticlinal walls are straight and angular (e.g. *C.camphora* and *C.glanduliferum*), or round (e.g. *C.bodinieri*, *C.glanduliferum* and *C.porrectum*), or sinuous (e.g. *C.iners* and *C.saxatile*), thickened (e.g. *C.daphnoides*, *C.randaiense*, and *C.subavenium*) or not (e.g. *C.camphora*, *C.longepaniculatum*, *C.migao*, and *C.porrectum*). Periclinal walls of epidermal cells are either smooth (e.g. *C.camphora*, *C.glanduliferum*, *C.longepaniculatum*, *C.migao*, and *C.porrectum*) or reticulate (e.g. *C.burmannii*, *C.iners*, and *C.randaiense*). The lower stomatal ledges of *Cinnamomum* under LM include different types, e.g. wide lip-shaped (e.g. *C.randaiense*, Fig. 7E), narrow lip-shaped (e.g. *C.migao*, Fig. 7C), bat-shaped (e.g. *C.longepaniculatum*, Fig. 7D), butterfly-shaped (e.g. *C.burmannii* and *C.daphnoides*, Fig. 7A, B, Table 3). The wide lip-shaped and narrow li-shaped sometimes concur in a certain species (e.g. *C.verum*, Fig. 7F). Stomatal surfaces under SEM possess at least five different types, i.e. circular (e.g. *C.chartophyllum*, Fig. 8A), eyelid-shaped (e.g. *C.tonkinense* and *C.jensenianum*, Fig. 8G and 8H), globose (e.g. *C.septentrionale* and *C.camphora*, Fig. 8E and 8F), lip-shaped (e.g. *C.longepaniculatum* and *C.migao*, Fig. 8C and 8D), and invisible when the stomata are densely covered with wax/appendages (Table 3).

### Phylogeny and character evolution

**Figure 9.**
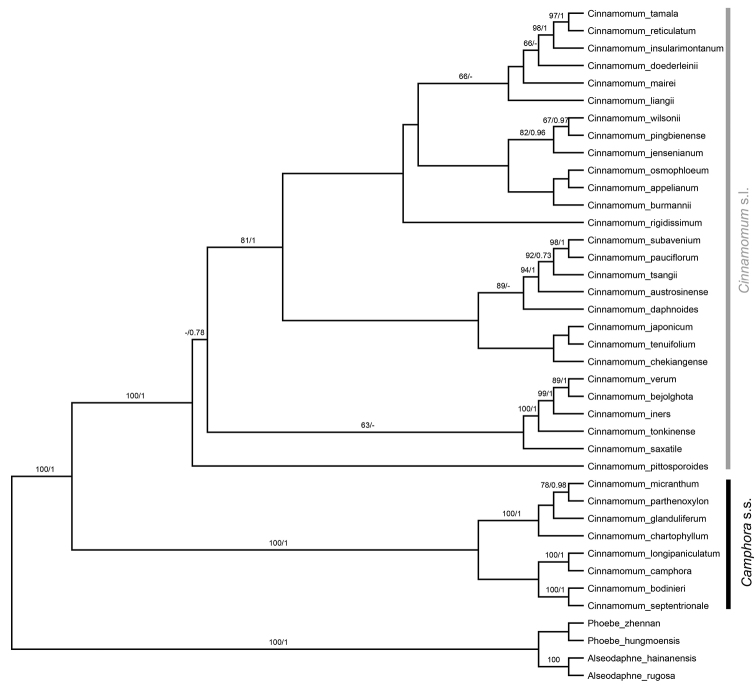
Phylogeny of the Asian *Cinnamomum* incorporating ML and BI trees. Upper number of the slash refers to the bootstrap value of the ML tree and the lower number of the slash refers to the posterior probabilities of the BI tree.

Asian *Cinnamomum* diverged into two robust clades (BS: 100; PP: 1.00, Fig. 9), one containing the species of sect. Camphora s.s. except *C.saxatile*, the other including the species of sect. Cinnamomum plus *C.saxatile*, which was previously ascribed to sect. Camphora. This latter clade is considered as sect. Cinnamomum s.l. here. However, relationships within the two clades were not completely resolved. A number of nodes were only poorly supported, bootstrap values were less than 50 and posterior probabilities were less than 0.70.

**Figure 10.**
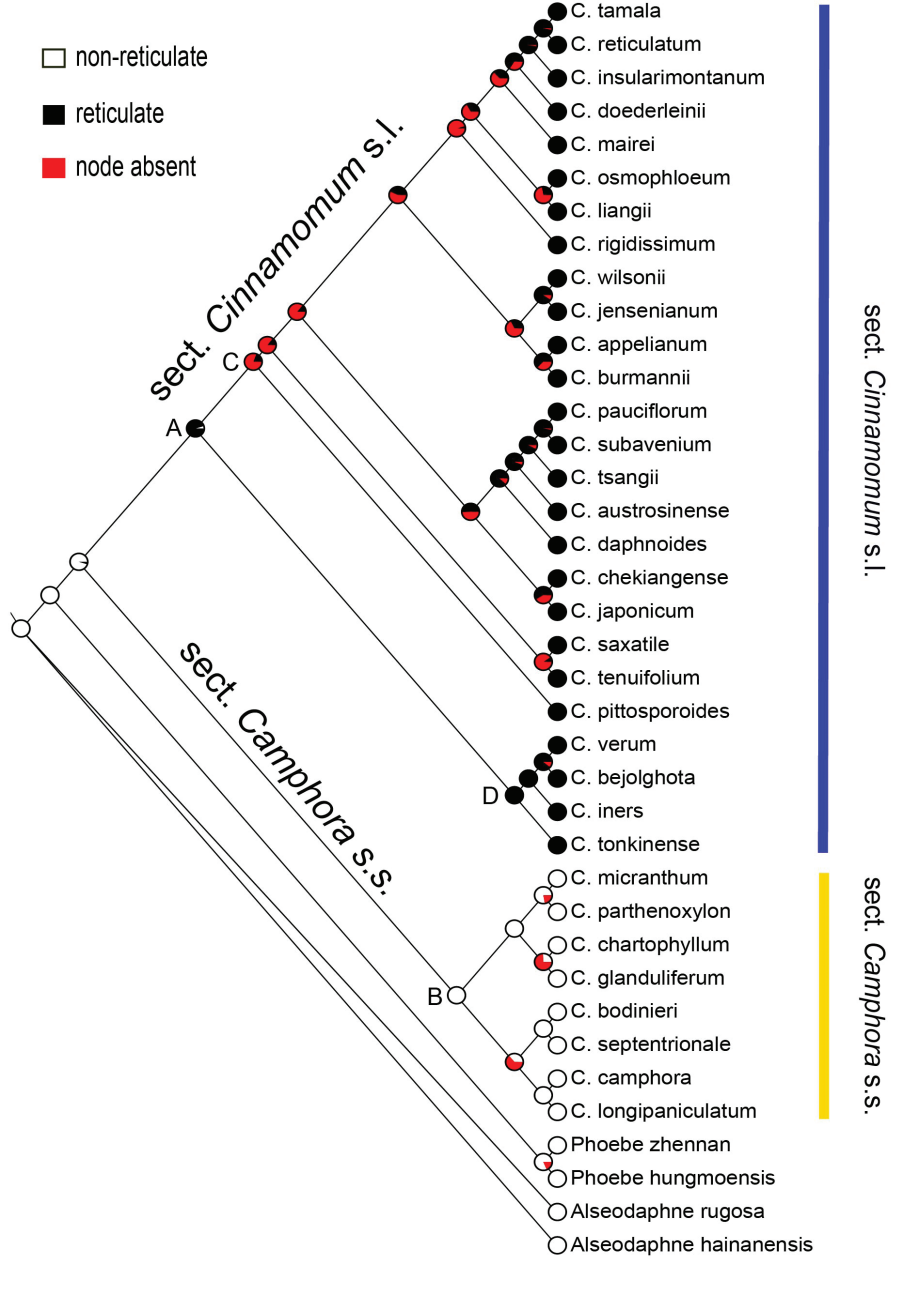
Ancestral character reconstruction of the periclinal wall reticulation by applying a ML tree block in Mesquite with a maximum likelihood approach and MK1 model. The common ancestor of Node A possesses reticulate periclinal wall with high likelihood (95.18%), and the ancestral Node B is reticulate with high likelihood (99.99%).

**Figure 11.**
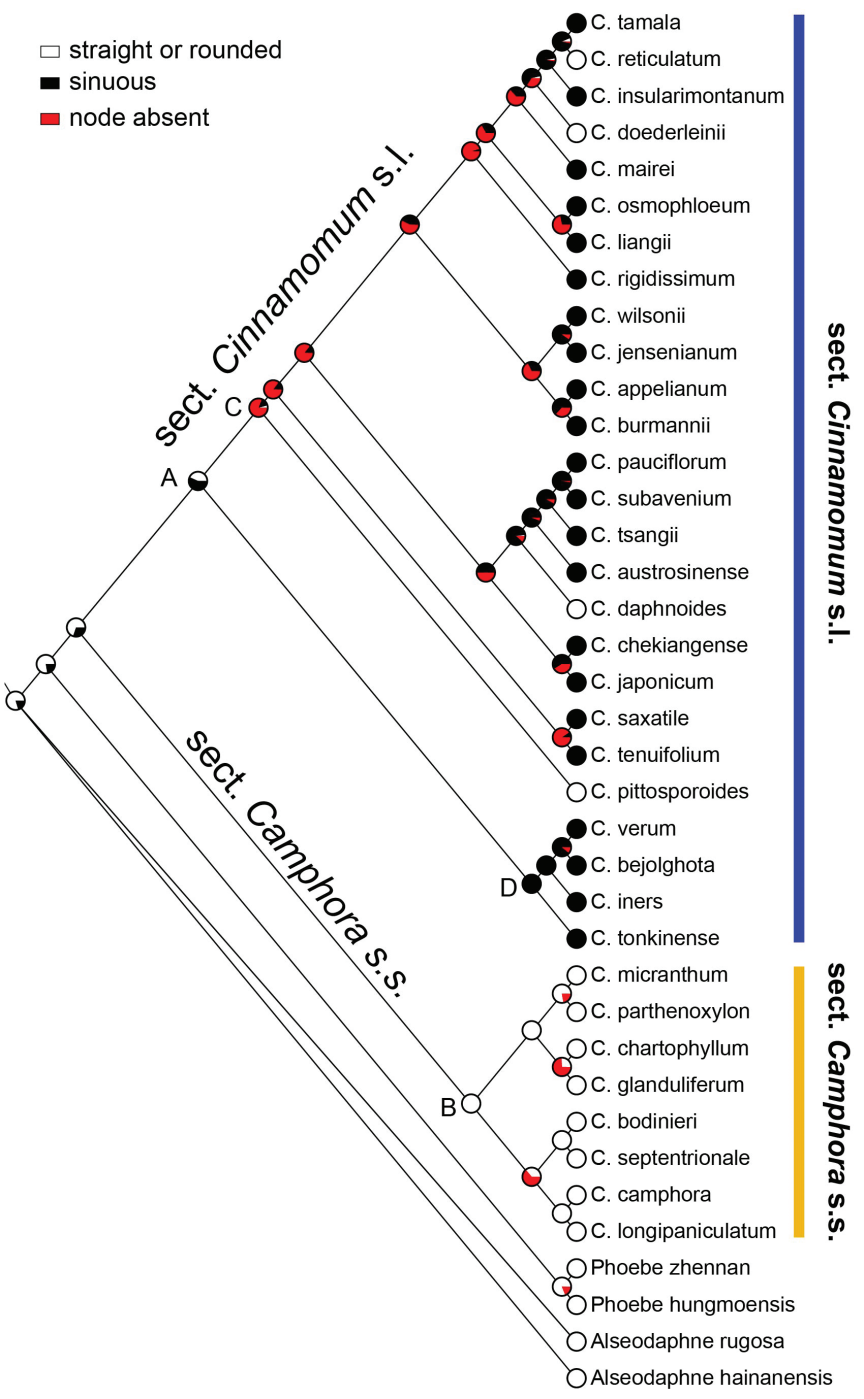
Ancestral character reconstruction of the epidermal cell shape and the straightness of anticlinal wall by applying a ML tree block in Mesquite with a maximum likelihood approach and MK1 model. Node A: the ancestral node of sect. Cinnamomum s.l. had sinuous anticlinal walls and irregular cell shapes or not, the probability being only 56.54%; Node B: the ancestral node of sect. Camphora s.s. possessed straight or curved anticlinal walls and polygonal cell shapes, the probability being 99.17%.

When simply mapping on the phylogenetic tree, the two character states of the periclinal wall reticulation allowed clear separation into two clades: non-reticulate for sect. Camphora s.s. and reticulate for sect. Cinnamomum s.l.; there is no overlap. However, neither epidermal cell shape nor anticlinal wall straightness are clear-cut. Sect. Cinnamomum s.l. usually possess sinuous anticlinal walls and irregular cell shapes, but a few species with straight or curved anticlinal walls and polygonal cell shapes were found to belong to this clade, e.g. *C.reticulatum*, *C.doederleinii*, *C.daphnoides*, and *C.pittosporoides*. Straight or curved anticlinal walls and polygonal cell shapes were common in sect. Camphora s.s., and we found no exception. For evolutionary history of periclinal wall reticulation (Fig. 10), the ancestral node A of sect. Cinnamomum s.l. was reticulate with high probability (95.18%), and the ancestral node B of sect. Camphora s.s. was non-reticulate with very high likelihood (99.99%). Anticlinal wall straightness and epidermal cell shape resulted in the same reconstruction (Fig. 11), the ancestral node of sect. Camphora s.s. possessed straight or curved anticlinal walls (node B), and polygonal cell shapes (99.17%), while it was uncertain whether the ancestral node of sect. Cinnamomum s.l. had sinuous anticlinal walls and irregular cell shapes or not, the probability being only 56.54% (node A).

## Discussion

In this study, leaf epidermal micromorphology of 48 species representing the two macromorphological sections of Asian *Cinnamomum* was studied. Our sampled species largely overlapped with the species sampling of the two recent molecular phylogenetic studies (Huang et al. 2016; Rohde et al. 2017), permitting an assessment of the systematic significance of leaf epidermal micromorphology within a phylogenetic context.

The polygonal to irregular epidermal cell shape and the straight to sinuous anticlinal walls have been described in previous reports (e.g. Christophel et al. 1996; Nishida and Christophel 1999; Nishida and van der Werff 2007, 2011; Yang et al. 2012; Trofimov and Rohwer 2018), but our study suggests that sect. Cinnamomum s.l. possesses an unusual reticulate periclinal wall which has not been studied carefully before in Lauraceae. We studied the reticulate periclinal wall under SEM, and hypothesize that uneven thickening of the periclinal wall gives rise to the reticulation under LM (Fig. 5). The reticulations of the periclinal wall are usually coarse in sect. Cinnamomum s.l., the spaces dividing the periclinal wall into reticulations are narrow. The reticulations in sect. Cinnamomum s.l. are rarely fine and appear to be ‘punctate’, e.g. in *C.iners* and *C.japonicum*, which is similar to that of a few species of *Beilschmiedia* Nees (Nishida and van der Werff 2007), where the spaces are wide. We prefer to describe the unusual periclinal wall in sect. Cinnamomum as reticulate but not punctate because they appear to be coarse but not dot-like.

Asian *Cinnamomum* species are classified into two sections according to the persistence of tepals, presence of perulate buds, leaf arrangement either alternate or subopposite, and leaf venation, i.e. sect. Camphora s.l. and sect. Cinnamomum s.s. (syn.: sect. Malabathrum Meisn. (1864: 10)). This classification was proposed by Meisner (1864) and followed by subsequent authors (e.g. Li et al. 1982). A recent phylogeny based on three nuclear sequences (ITS, *RPB*2 and *LEAFY*) suggests that a few taxa placed in sect. Camphora based on macromorphological characters actually belong to the clade consisting mainly of sect. Cinnamomum s.l., namely *C.saxatile*, *C.longipetiolatum* and an unidentified sample (Huang et al. 2016). Sect. Camphora s.s. is characterized by alternate, pinnately veined or weakly tripliveined leaves, mostly perulate buds and presence of domatia in the axils of lateral veins. However, these features occur also in sect. Cinnamomum s.l. when the clade includes *C.saxatile* and *C.longipetiolatum*. As a result, the current definition of both sect. Cinnamomum s.l. and *Camphora* s.s. using presence or absence of these morphological characters is problematic.

Our study suggests that the leaf epidermal micromorphology can be divided into two different types and the two types of leaf epidermal micromorphology are surprisingly congruent with the clades retrieved in the analysis of Huang et al. (2016): the taxa of sect. Camphora s.s. possess the smooth upper epidermis, while those of sect. Cinnamomum have the reticulate upper epidermis. The reticulate type of periclinal walls is derived, because this type has not been found in any other groups of the family (see Christophel et al. 1996; Nishida and Christophel 1999; Nishida and van der Werff 2007, 2011, 2014; Yang et al. 2012; Zeng et al. 2014; Nishida et al. 2016). In the character reconstruction, the ancestral node of the Asian *Cinnamomum* possesses a non-reticulate type of periclinal walls with high probability (95.34%). The two types of periclinal walls are clade-specific (Fig. 10), and the reticulate type appears to have originated in the ancestor of sect. Cinnamomum s.l. The reticulate type is shared by sect. Cinnamomum s.l. and its ancestor with a probability of 95.18%, and the non-reticulate type is shared by sect. Camphora s.s. and its ancestor with a probability of 100%. We consider that the reticulate type of periclinal walls is a synapomorphy of sect. Cinnamomum s.l., and is useful in classification of the two clades.

Both leaf epidermal cell shape and the straightness of anticlinal walls are not clade specific and transitional between the two groups/clades of Asian *Cinnamomum* (Fig. 11). In sect. Cinnamomum s.l., a few species possess polygonal epidermal cell shape and straight/curved anticlinal walls, which are common in sect. Camphora s.s., e.g. *C.daphnoides*, *C.doederleinii*, *C.pittosporoides*, *C.reticulatum*, and *C.scortechinii*. These species possess opposite triveined/tripliveined leaves. A few other species were not examined in phylogenetic studies, but they too possess reticulate periclinal walls, and opposite/subopposite, triveined/tripliveined leaves lacking domatia, viz. *C.liangii*, *C.litseifolium*, *C.macrostemon*, *C.pseudopedunculatum*, *C.tetragonum*, *C.validinerve*, and *C.ovalifolium*. We thus expect them to belong to sect. Cinnamomum s.s. Another two species examined here, *C.ilicioides* and *C.migao* possess non-reticulate periclinal walls, polygonal cell shape, straight/rounded anticlinal walls, perulate buds, and alternate and penninerved leaves which bear inconspicuous domatia in axils of lateral veins, so they clearly belong to sect. Camphora s.s.

Phylogenetic relationships of *Sassafras* have not been resolved. Rohde et al. (2017) gave conflicting phylogenetic results on *Sassafras* based on nrDNA and cpDNA sequences. The phylogeny based on nrITS indicates that *Sassafras* is sister to the *Cinnamomum*+*Aiouea*+*Ocotea* complex, nevertheless, the phylogeny based on cpDNA suggests that *Sassafras* forms a clade together with two species of sect. Camphora, i.e. *C.bodinieri* and *C.glanduliferum*, making the sect. Camphora polyphyletic. Trofimov and Rohwer (2020) indicated that Sassafras is sister to sect. Camphora based on nrITS and *psb*A-*trn*H. Liu et al. (2021) reported conflicts between nuclear and plastid phylogenetic results. In their analysis, Sassafras is either sister to sect. Camphora (nrDNA phylogeny) or to a clade consisting of *C.caudiferum* and *C.porrectum* (plastome phylogeny), but their result on *Sassafras* is not conclusive due to poor sampling of *Cinnamomum*. Our leaf anatomy indicates that *Sassafras* does possess Type I upper leaf epidermis as in sect. Camphora (and most other Lauraceae), i.e. polygonal epidermal cells, straight anticlinal walls, and non-reticulate periclinal walls.

The genus *Cinnamomum* was formerly considered to be amphi-Pacific (Rohwer 1993; Lorea-Hernandez 1996; van der Werff 2001), but a recent phylogenetic study (Rohde et al. 2017) suggested that the American species are closer to the likewise predominantly American *Ocotea* complex than to Asian *Cinnamomum*; they have now been accommodated in *Aiouea* (Rohde et al. 2017). The Old World *Cinnamomum* is thus a diphyletic group, and includes two clades (Huang et al. 2016; Rohde et al. 2017, and this study). Sect. Cinnamomum appears to be sister to the Neotropical clade in Huang et al. (2016) but it is the sect. Camphora that appears to be sister to the Neotropical clade in nrITS analysis of Rohde et al. (2017). Whichever is correct, the Asian *Cinnamomum* is not a monophyletic group and should be further subdivided into two genera. Our new study clearly suggests that use of leaf epidermal micromorphological characters leads to the recognition of two distinct groups that are clade-specific and highly predictive. We thus provide micromorphological support to classify the Asian *Cinnamomum* into two genera.

## Supplementary materials

Supplementary material 1 Phylogenetic trees of *Cinnamomum* and sequences obtained from the GenBank Data type Docx file. Explanation note Phylogenetic trees. https://binary.pensoft.net/file/594745This dataset is made available under the Open Database License (http://opendatacommons.org/licenses/odbl/1.0/). The Open Database License (ODbL) is a license agreement intended to allow users to freely share, modify, and use this Dataset while maintaining this same freedom for others, provided that the original source and author(s) are credited.Zeng Gang, Bing Liu, Jens G. Rohwer, David Kay Ferguson, Yong Yang
