# Supplementary material for: Leaf epidermal micromorphology defining the clades in Cinnamomum (Lauraceae)
Source: PhytoKeys. 2021 Oct 4;182:125–48. doi: 10.3897/phytokeys.182.67289 (PMC8516828; doi:10.3897/phytokeys.182.67289)
Supplement: Supplementary material 1 — Phylogenetic trees of Cinnamomum and sequences obtained from the GenBank [file phytokeys-182-125-s001.docx]

**Supplementary Materials**

**
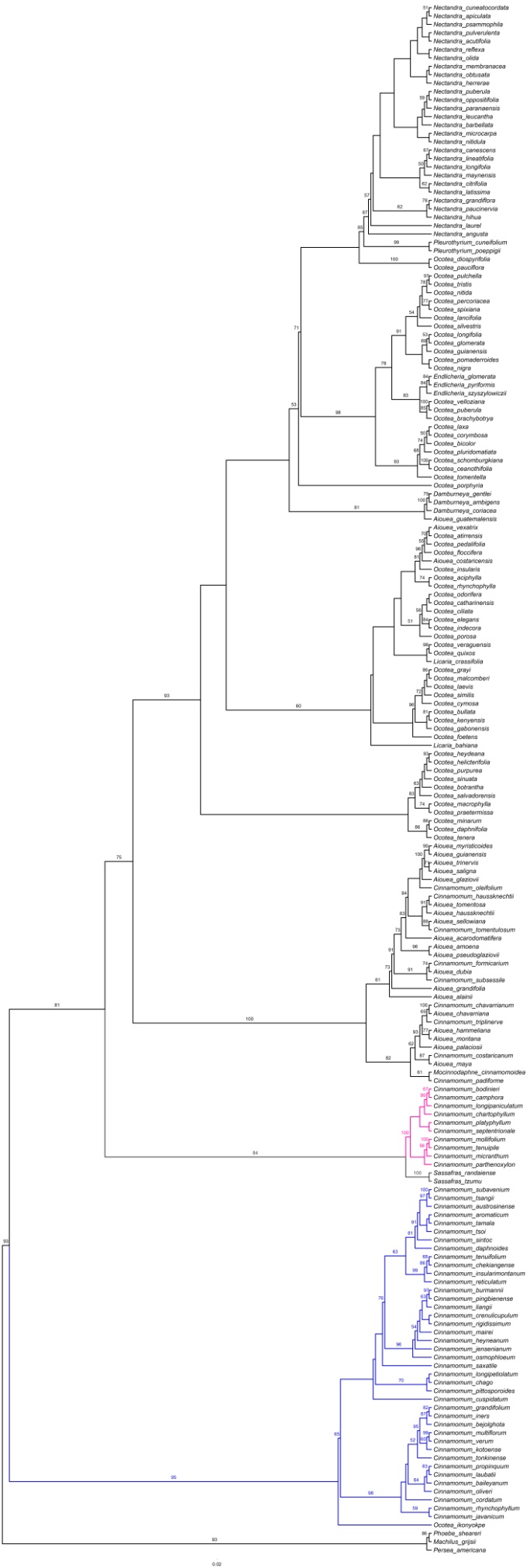
**

**Figure S1**. A maximum likelihood cladogram of the *Cinnamomum* group based on nrITS displaying the polyphyletic status of the Asian *Cinnamomum*. Sequences were retrieved from Genbank, and their accession number was listed in Supplementary Materials-Table S1. Sequences were aligned in Mafft 7.308-win64, and manually adjusted in BioEdit 7.2.5. The format was transformed in SequenceMatrix Windows 1.7.8. The final alignment of nrITS contains 185 sequences with 751 columns, 471 distinct patterns, 226 parsimony-informative, 117 singleton sites, and 408 constant sites. The tree was reconstructed with RAxML-HPC2 on XSEDE with the GTRCAT model. Bootstrap values were only marked above branches when they are higher than 50%. The final tree was viewed and adjusted in FigTree v1.4.0, and then beautified in Adobe Illustrator CS2 ver. 12.0.0. We also reconstructed a ML tree using IQ-TREE Web Server (<http://iqtree.cibiv.univie.ac.at/>) with bootstrap analysis as ‘standard’ and the number of bootstrap alignments as ‘100’, and got the same topology with low to moderate (60% or higher) bootstrap supports in general.

**
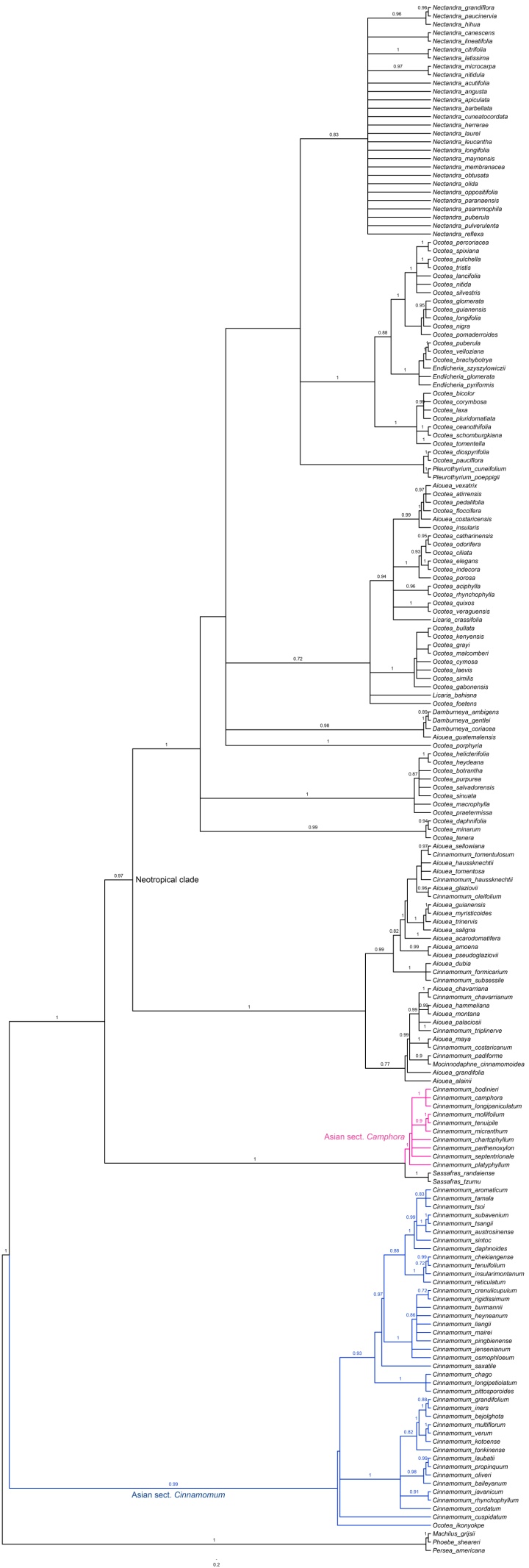
**

**Figure S2**. A Bayesian cladogram of the *Cinnamomum* group based on nrITS displaying the polyphyletic status of the Asian *Cinnamomum*. Sequences were retrieved from Genbank, and their accession number was listed in Supplementary-Table S1. Sequences were aligned in Mafft 7.308-win64, and manually adjusted in BioEdit 7.2.5. The format was transformed in SequenceMatrix Windows 1.7.8. The tree was reconstructed with MrBayes on XSEDE with the Nst=6, Rates=invgamma, Ngen=1,000,000, Markov chain was sampled every 1,000, and Sumt Burnin Value=25. The final tree was viewed and adjusted in FigTree v1.4.0, and then beautified in Adobe Illustrator CS2 ver. 12.0.0. Posterior probabilities were shown above branches only when they are higher than 0.70.

**Table S1**. Sequences obtained from Genbank for phylogenetic reconstruction of the *Cinnamomum* group.

| Species | Accession No. |
| --- | --- |
| *Aiouea acarodomatifera* Kosterm. | MF110006.1 |
| *Aiouea alainii* (C.K.Allen) R.Rohde | MF110007.1 |
| *Aiouea amoena* (Nees & Mart) R.Rohde | MF110008.1 |
| *Aiouea chavarriana* (Hammel) R.Rohde | MF110009.1 |
| *Aiouea costaricensis* (Mez) Kosterm. | MF110011.1 |
| *Aiouea dubia* (Kunth) Mez | KU139905.1 |
| *Aiouea glaziovii* (Mez) R.Rohde | MF110013.1 |
| *Aiouea grandifolia* van der Werff | MF110014.1 |
| *Aiouea guatemalensis* (Lundell) Renner | MF110015.1 |
| *Aiouea guianensis* Aubl. | AF272251.1 |
| *Aiouea hammeliana* (W.C.Burger) R.Rohde | MF110016.1 |
| *Aiouea haussknechtii* (Mez) R.Rohde | MF110017.1 |
| *Aiouea maya* Lorea-Hern. | MF110020.1 |
| *Aiouea montana* (Sw.) R.Rohde | MF110021.1 |
| *Aiouea myristicoides* Mez | MF110022.1 |
| *Aiouea palaciosii* (van der Werff) R.Rohde | MF110023.1 |
| *Aiouea pseudoglaziovii* Lorea-Hern. | MF110024.1 |
| *Aiouea saligna* Meisn. | KX509821.1 |
| *Aiouea sellowiana* (Nees & Mart) R.Rohde | MF110025.1 |
| *Aiouea tomentosa* (Meisn.) R.Rohde | MF110031.1 |
| *Aiouea trinervis* Meisn. | MF110032.1 |
| *Aiouea vexatrix* van der Werff | MF110033.1 |
| *Cinnamomum austrosinense* H.T.Chang | KU139818.1 |
| *Cinnamomum baileyanum* (F.Muell. ex F.M.Bailey) Francis | KU139820.1 |
| *Cinnamomum bejolghota* (Buch.-Ham.) Sweet | KU139822.1 |
| *Cinnamomum bodinieri* H.Lév. | KU139824.1 |
| *Cinnamomum burmannii* (Nees & T.Nees) Blume | KU139825.1 |
| *Cinnamomum camphora* (L.) J.Presl | KX509822.1 |
| *Cinnamomum chago* B.S.Sun & H.L.Zhao | KU139830.1 |
| *Cinnamomum chartophyllum* H.W.Li | KU139831.1 |
| *Cinnamomum chavarrianum* (Hammel) Kosterm. | KU139833.1 |
| *Cinnamomum chekiangense* Nakai | KU139834.1 |
| *Cinnamomum cordatum* Kosterm. | KU139835.1 |
| *Cinnamomum costaricanum* (Mez & Pittier) Kosterm. | KU139836.1 |
| *Cinnamomum crenulicupulum* Kosterm. | KU139838.1 |
| *Cinnamomum cuspidatum* Miq. | KU139839.1 |
| *Cinnamomum daphnoides* Siebold & Zucc. | KU139841.1 |
| *Cinnamomum dubium* Nees | KU139862.1 |
| *Cinnamomum formicarium* van der Werff & Lorea-Hern. | KX509823.1 |
| *Cinnamomum grandifolium* Cammerl. | KU139844.1 |
| *Cinnamomum haussknechtii* (Mez) Kosterm. | KU139845.1 |
| *Cinnamomum heyneanum* Nees | KU139847.1 |
| *Cinnamomum iners* Reinw. ex Blume | KU139848.1 |
| *Cinnamomum insularimontanum* Hayata | KX509825.1 |
| *Cinnamomum javanicum* Blume | KU139852.1 |
| *Cinnamomum jensenianum* Hand.-Mazz. | KU139853.1 |
| *Cinnamomum kotoense* Kaneh. & Sasaki | KU139854.1 |
| *Cinnamomum laubatii* F.Muell. | KU139855.1 |
| *Cinnamomum liangii* C.K.Allen | KU139856.1 |
| *Cinnamomum longipaniculatum* (Gamble) N.Chao ex H.W.Li | KU139857.1 |
| *Cinnamomum longipetiolatum* H.W.Li | KU139858.1 |
| *Cinnamomum mairei* H.Lév. | KU139859.1 |
| *Cinnamomum micranthum* (Hayata) Hayata | KU139860.1 |
| *Cinnamomum mollifolium* H.W.Li | KU139861.1 |
| *Cinnamomum oleifolium* (Mez) Kosterm. | KU139864.1 |
| *Cinnamomum oliveri* F.M.Bailey | KU139865.1 |
| *Cinnamomum osmophloeum* Kaneh. | KU139866.1 |
| *Cinnamomum padiforme* (Standl. & Steyerm.) Kosterm. | KU139868.1 |
| *Cinnamomum parthenoxylon* (Jack) Meisn. | KU139871.1 |
| *Cinnamomum pingbienense* H.W.Li | KU139873.1 |
| *Cinnamomum pittosporoides* Hand.-Mazz. | KU139874.1 |
| *Cinnamomum platyphyllum* (Diels) C.K.Allen | KU139875.1 |
| *Cinnamomum porrectum* (Roxb.) Kosterm. | KU139870.1 |
| *Cinnamomum propinquum* F.M.Bailey | KU139876.1 |
| *Cinnamomum reticulatum* Hayata | KU139879.1 |
| *Cinnamomum rhynchophyllum* Miq. | KU139880.1 |
| *Cinnamomum rigidissimum* H.T.Chang | KU139881.1 |
| *Cinnamomum saxatile* H.W.Li | KU139882.1 |
| *Cinnamomum septentrionale* Hand.-Mazz. | KU139883.1 |
| *Cinnamomum sintoc* Blume | KU139884.1 |
| *Cinnamomum subavenium* Miq. | KU139888.1 |
| *Cinnamomum subsessile* (Meisn.) Kosterm. | KU139889.1 |
| *Cinnamomum tamala* (Buch.-Ham.) T.Nees & Eberm. | KU139891.1 |
| *Cinnamomum tenuifolium* (Makino) Sugim. | KU139892.1 |
| *Cinnamomum tenuipile* Kosterm. | KU139893.1 |
| *Cinnamomum tomentulosum* Kosterm. | KU139894.1 |
| *Cinnamomum tonkinense* (Lecomte) A.Chev. | KU139896.1 |
| *Cinnamomum triplinerve* (Ruiz & Pav.) Kosterm. | KU139897.1 |
| *Cinnamomum tsangii* Merr. | KU139900.1 |
| *Cinnamomum tsoi* C.K.Allen | KU139901.1 |
| *Cinnamomum verum* J.Presl | KU139827.1 |
| *Damburneya ambigens* (S.F.Blake) Trofimov | KX509828.1 |
| *Damburneya coriacea* (Sw.) Trofimov & Rohwer | KX509829.1 |
| *Damburneya gentlei* (Lundell) Trofimov | KX509830.1 |
| *Endlicheria glomerata* Mez | MF110065.1 |
| *Endlicheria pyriformis* (Nees) Mez | MF110066.1 |
| *Endlicheria szyszylowiczii* Mez | MF110067.1 |
| *Licaria bahiana* Kurz | MF110068.1 |
| *Licaria crassifolia* (Poir.) P.L.R. de Moraes | MF110069.1 |
| *Machilus grijsii* Hance | KX509833.1 |
| *Mocinnodaphne cinnamomoidea* Lorea-Hern. | AF272288.1 |
| *Nectandra acutifolia* (Ruiz & Pav.) Mez | KX509834.1 |
| *Nectandra angusta* Rohwer | KX509835.1 |
| *Nectandra apiculata* Rohwer | KX509836.1 |
| *Nectandra barbellata* Coe-Teix. | KX509837.1 |
| *Nectandra canescens* Nees & Mart. | KX509838.1 |
| *Nectandra citrifolia* Mez & Rusby | KX509842.1 |
| *Nectandra cuneatocordata* Mez | KX509843.1 |
| *Nectandra grandiflora* Nees & Mart. | KX509845.1 |
| *Nectandra herrerae* O.C.Schmidt | KX509846.1 |
| *Nectandra hihua* (Ruiz & Pav.) Rohwer | KX509847.1 |
| *Nectandra latissima* Rohwer | KX509848.1 |
| *Nectandra laurel* Klotzsch ex Nees | KX509849.1 |
| *Nectandra leucantha* Nees & Mart. | KX509850.1 |
| *Nectandra lineatifolia* (Ruiz & Pav.) Mez | KX509851.1 |
| *Nectandra longifolia* (Ruiz & Pav.) Nees | KX509852.1 |
| *Nectandra maynensis* Mez | KX509853.1 |
| *Nectandra membranacea* (Sw.) Griseb. | KX509854.1 |
| *Nectandra microcarpa* Meisn. | KX509856.1 |
| *Nectandra nitidula* Nees & Mart. | KX509857.1 |
| *Nectandra obtusata* Rohwer | KX509858.1 |
| *Nectandra olida* Rohwer | KX509859.1 |
| *Nectandra oppositifolia* Nees & Mart. | KX509860.1 |
| *Nectandra paranaensis* Coe-Teix. | KX509861.1 |
| *Nectandra paucinervia* Coe-Teix. | KX509862.1 |
| *Nectandra psammophila* Nees & C. Mart. | MF110070.1 |
| *Nectandra puberula* (Schott) Nees | KX509863.1 |
| *Nectandra pulverulenta* Nees | KX509864.1 |
| *Nectandra reflexa* Rohwer | KX509865.1 |
| *Ocotea aciphylla* (Nees & Mart.) Mez | KX509866.1 |
| *Ocotea atirrensis* Mez & Donn. | MF110077.1 |
| *Ocotea botrantha* Rohwer | AF272297.1 |
| *Ocotea brachybotrya* (Meisn.) Mez | GQ480376.1 |
| *Ocotea bullata* (Burch.) E. Meyer | AF272298.1 |
| *Ocotea catharinensis* Mez | KF420963.1 |
| *Ocotea ceanothifolia* (Nees) Mez | AF272299.1 |
| *Ocotea ciliata* L.C.S. Assis & Mello-Silva | MF110072.1 |
| *Ocotea corymbosa* (Meisn.) Mez | GQ480375.1 |
| *Ocotea cymosa* (Nees) Palacky | KJ189018.1 |
| *Ocotea daphnifolia* (Meisn.) Mez | GQ480378.1 |
| *Ocotea diospyrifolia* (Meisn.) Mez | GQ480379.1 |
| *Ocotea foetens* (Aiton) Baill. | KX509869.1 |
| *Ocotea gabonensis* Fouilloy | MF110075.1 |
| *Ocotea glomerata* (Nees) Mez | GQ480380.1 |
| *Ocotea grayi* van der Werff | AF272301.1 |
| *Ocotea guianensis* Aubl. | AF272302.1 |
| *Ocotea helicterifolia* (Meisn.) Hemsl. | AF272303.1 |
| *Ocotea heydeana* (Mez & Donn.Sm.) Bernardi | AF272304.1 |
| *Ocotea ikonyokpe* van der Werff | AF272305.1 |
| *Ocotea indecora* (Schott) Mez | MF110076.1 |
| *Ocotea insularis* (Meisn.) Mez | MF110074.1 |
| *Ocotea kenyensis* (Chiov.) Robyns & R.Wilczek | KJ189025.1 |
| *Ocotea lancifolia* (Schott) Mez | GQ480383.1 |
| *Ocotea laxa* (Nees) Mez | GQ480384.1 |
| *Ocotea longifolia* Kunth | GQ480385.1 |
| *Ocotea macrophylla* Kunth | KX509870.1 |
| *Ocotea malcomberi* van der Werff | AF272307.1 |
| *Ocotea minarum* (Nees & Mart.) Mez | GQ480386.1 |
| *Ocotea nigra* Benoist | AF272308.1 |
| *Ocotea nitida* (Meisn.) Rohwer | GQ480387.1 |
| *Ocotea odorifera* (Vell.) Rohwer | KX509871.1 |
| *Ocotea pauciflora* (Nees) Mez | AF272310.1 |
| *Ocotea percoriacea* Kosterm. | AF272311.1 |
| *Ocotea pluridomatiata* A.Quinet | GQ480389.1 |
| *Ocotea pomaderroides* (Meisn.) Mez | GQ480390.1 |
| *Ocotea porosa* (Nees & Mart.) Barroso | MF110078.1 |
| *Ocotea porphyria* (Griseb.) van der Werff | MF110079.1 |
| *Ocotea praetermissa* van der Werff | KX509872.1 |
| *Ocotea puberula* (Rich.) Nees | GQ480391.1 |
| *Ocotea pulchella* (Nees & Mart.) Mez | KX509873.1 |
| *Ocotea purpurea* (Mez) van der Werff | KX509874.1 |
| *Ocotea quixos* (Lam.) Kosterm. | MF110080.1 |
| *Ocotea racemosa* (Danguy) Kosterm. | KJ189017.1 |
| *Ocotea salvadorensis* (Lundell) van der Werff | KX509875.1 |
| *Ocotea schomburgkiana* (Nees) Mez | AF272315.1 |
| *Ocotea silvestris* Vattimo | GQ480394.1 |
| *Ocotea sinuata* (Mez) Rohwer | KX509876.1 |
| *Ocotea spixiana* (Nees) Mez | AF272316.1 |
| *Ocotea tenera* Mez & Donn. | MF110082.1 |
| *Ocotea tomentella* Sandwith | AF272317.1 |
| *Ocotea tristis* (Nees & Mart.) Mez | AF272318.1 |
| *Ocotea velloziana* (Meisn.) Mez | GQ480395.1 |
| *Ocotea veraguensis* (Meisn.) Mez | AF272319.1 |
| *Persea americana* Mill. | KX509877.1 |
| *Phoebe sheareri* (Hemsl.) Gamble | KX509878.1 |
| *Pleurothyrium cuneifolium* Nees | KX509879.1 |
| *Pleurothyrium poeppigii* Nees | KX509880.1 |
| *Sassafras randaiense* (Hayata) Rehder | EF491212.1 |
| *Sassafras tzumu* (Hemsl.) Hemsl. | EF491209.1 |

**Table S2**. Sequences obtained from the GenBank for phylogeny of Asian *Cinnamomum*.

| Taxon | ITS | RPB2 | LEAFY |
| --- | --- | --- | --- |
| *Alseodaphnopsis hainanensis* | FJ755440 | KU140409 | HQ697006 |
| *Alseodaphnopsis rugosa* | HQ697183 | KU140410 | HQ697012 |
| *Cinnamomum appelianum* | KU139817 | KU140330 | KU140244 |
| *Cinnamomum austrosinense* | KU139818 | KU140331 | KU140245 |
| *Cinnamomum bejolghota* | KU139822 | KU140335 | KU140249 |
| *Cinnamomum bodinieri* | KU139824 | KU140336 | KU140251 |
| *Cinnamomum burmannii* | KU139825 | KU140337 | KU140252 |
| *Cinnamomum camphora* | KU139826 | KU140338 | KU140253 |
| *Cinnamomum chartophyllum* | KU139832 | KU140344 | KU140259 |
| *Cinnamomum chavarrinum* | AF272261 | KU140345 | − |
| *Cinnamomum chekiangense* | MF110041 | KU140346 | KU140260 |
| *Cinnamomum daphnoides* | FM957803 | KU140352 | KU140266 |
| *Cinnamomum doederleinii* | KU139842 | − | KU174408 |
| *Cinnamomum glanduliferum* | KU139843 | KU140354 | KU140269 |
| *Cinnamomum iners* | KU139849 | KU140360 | KU140275 |
| *Cinnamomum insularimontanum* | KY271510 | KU140361 | KU174418 |
| *Cinnamomum japonicum* | KU139851 | KU140361 | KU140277 |
| *Cinnamomum jensenianum* | KU139853 | KU140363 | KU140279 |
| *Cinnamomum liangii* | KU139856 | KU140366 | KU174422 |
| *Cinnamomum longipaniculatum* | KX546754 | KT248715 | KU140283 |
| *Cinnamomum macrostemon* | GU598521 | − | − |
| *Cinnamomum mairei* | KU139859 | KU140368 | KU174423 |
| *Cinnamomum micranthum* | KY271519 | KU140369 | GQ260581 |
| *Cinnamomum osmophloeum* | KY271528 | KU140375 | − |
| *Cinnamomum parthenoxylon* | KU139871 | KU140377 | KU140295 |
| *Cinnamomum pauciflorum* | KU139872 | KU140378 | KU140296 |
| *Cinnamomum pingbienense* | KU139873 | KU140379 | KU140297 |
| *Cinnamomum pittosporoides* | KU139874 | KU140380 | KU140298 |
| *Cinnamomum reticulatum* | KU139879 | − | KU174432 |
| *Cinnamomum rigidissimum* | KU139881 | KU140386 | KU140305 |
| *Cinnamomum saxatile* | KU139882 | KU140387 | KU140306 |
| *Cinnamomum septentrionale* | KU139883 | KU140388 | KU140307 |
| *Cinnamomum subavenium* | KU139888 | KU140393 | KU140312 |
| *Cinnamomum tamala* | KX822090 | KU140396 | KU174439 |
| *Cinnamomum tenuifolium* | KU139892 | KU140397 | KU140316 |
| *Cinnamomum tenuifolium* | − | − | KU140316 |
| *Cinnamomum tonkinense* | KU139895 | KU140400 | KU140319 |
| *Cinnamomum tsangii* | KU139900 | KU140405 | KU140324 |
| *Cinnamomum verum* | MF110061 | KU140407 | KU140326 |
| *Cinnamomum wilsonii* | KU139904 | KU140408 | KU140328 |
| *Phoebe hungmoensis* | HQ697206 | KU140413 | HQ697138 |
| *Phoebe zhennan* | HQ697212 | KT248761 | HQ697161 |
